# Chip Formation and Orthogonal Cutting Optimisation of Unidirectional Carbon Fibre Composites

**DOI:** 10.3390/polym15081897

**Published:** 2023-04-15

**Authors:** Alessandro Abena, Sein Leung Soo, Sabbah Ataya, Hany Hassanin, Mahmoud Ahmed El-Sayed, Mahmoud Ahmadein, Naser A. Alsaleh, Mohamed M. Z. Ahmed, Khamis Essa

**Affiliations:** 1 School of Engineering, University of Birmingham, Birmingham B15 2TT, UK; 2 Department of Mechanical Engineering, Imam Mohammad Ibn Saud Islamic University (IMSIU), Riyadh 11432, Saudi Arabia; 3 School of Engineering, Technology, and Design, Canterbury Christ Church University, Canterbury CT1 1QU, UK; 4 Department of Industrial and Management Engineering, Arab Academy for Science, Technology and Maritime Transport, Alexandria 21599, Egypt; 5 Department of Production Engineering and Mechanical Design, Tanta University, Tanta 31512, Egypt; 6 Mechanical Engineering Department, College of Engineering at Al Kharj, Prince Sattam Bin Abdulaziz University, Al Kharj 16273, Saudi Arabia; moh.ahmed@psau.edu.sa

**Keywords:** UD-CFRP, orthogonal cutting, bounce back, cutting edge, chip formation

## Abstract

This study presents a thorough experimental investigation utilising the design of experiments and analysis of variance (ANOVA) to examine the impact of machining process parameters on chip formation mechanisms, machining forces, workpiece surface integrity, and damage resulting from the orthogonal cutting of unidirectional CFRP. The study identified the mechanisms behind chip formation and found it to significantly impact the workpiece orientation of fibre and the tool’s cutting angle, resulting in increased fibre bounceback at larger fibre orientation angles and when using smaller rake angle tools. Increasing the depth of cut and fibre orientation angle results in an increased damage depth, while using higher rake angles reduces it. An analytical model based on response surface analysis for predicting machining forces, damage, surface roughness, and bounceback was also developed. The ANOVA results indicate that fibre orientation is the most significant factor in machining CFRP, while cutting speed is insignificant. Increasing fibre orientation angle and depth leads to deeper damage, while larger tool rake angles reduce damage. Machining workpieces with 0° fibre orientation angle results in the least subsurface damage, and surface roughness is unaffected by the tool rake angle for fibre orientations between 0° to 90° but worsens for angles greater than 90°. Optimisation of cutting parameters was subsequently carried out to improve machined workpiece surface quality and reduce forces. The experimental results showed that negative rake angle and cutting at moderately low speeds (366 mm/min) are the optimal conditions for machining laminates with a fibre angle of *θ* = 45°. On the other hand, for composite materials with fibre angles of *θ* = 90° and *θ* = 135°, it is recommended to use a high positive rake angle and cutting speeds.

## 1. Introduction

The use of reinforced plastic composites has steadily increased in the automotive and aerospace manufacturing industries. Composite materials offer excellent strength-to-weight ratio and corrosion resistance, making them popular. The need for lightweight aircraft and automobile structures, driven by strict CO_2_ emission regulations, has further increased the use of composites [1,2].

On the other hand, hybrid materials that combine composites with plastic or metal, such as steel plate cold commercial (SPCC), are being utilised in automotive structures to improve crash load performance. One of the benefits of using these materials is that they reduce the weight of composites, specifically during a crash. Carbon fibre reinforced polymer (CFRP) is a highly versatile material widely used across various applications thanks to its high specific strength, fatigue, corrosion, and electrical insulating properties. It is particularly valued for its ability to create lightweight structures [3].

Composite products are commonly produced using near-net-shape forming techniques, which can help reduce material waste and production time. The properties of CFRP typically depend on various parameters, such as fibre orientation and curing conditions [4]. However, to achieve the necessary precision and accuracy, additional machining processes may be required to create features, such as holes and slots that are critical for assembly and meet the required dimensional tolerances. However, such operations can induce flaws both at the micro and macro level (e.g., delamination, debonding, matrix and fibre fracture, etc.). Consequently, minimising defects throughout machining is important to reduce the risk that the integrity and performance of the component are compromised during service.

There have been several research studies conducted to investigate the impact of fibre orientation on machinability. However, most of the earlier research has primarily focused on performing an orthogonal cut on composites that are made of uni-directional carbon fibre reinforced polymers (CFRP). The relationship between the orientation of fibres within composite materials and the resultant machining forces has been thoroughly explored and documented in the existing literature [5,6,7]. The cutting force gradually increases until it reaches a critical fibre angle (*θ*) between 75° to 90°, beyond which there is an abrupt increase followed by a decrease. The thrust force varies with fibre orientation, with the highest value between 0° to 90° and the lowest when *θ* is greater than 90°. Wang and Zhang [6] and Kaneeda et al. [8] studied the effect of the tool rake angle on the cutting and thrust forces. They found that the direction of thrust force was reversed during machining when using tools with large rake angles and large fibre orientation of 90° to 120°. Conversely, Wang et al. [5] demonstrated that although the clearance angle had an insignificant effect on cutting forces, the thrust force decreased with the increase in the tool clearance angle. The reduction in thrust forces is due to the elastic recovery of the component after machining, known as bounce back. Chips formed during CFRP machining were studied, with rake angle and fibre orientation as significant factors. Five chip formation mechanisms were identified and described by Wang et al. using a sharp cutting tool with a radius smaller than the fibre diameter [5]. These mechanisms were also confirmed by several other publications [9,10,11]. The impact of rounded cutting edges at the microscale level has received limited attention in research studies, resulting in a relatively scarce understanding of this aspect in the field of machining. A different chip formation mechanism was observed when machining workpieces having a fibre angle of *θ* = 90° using a rounded cutting edge [12]. The tool did not function like a sharp edge and was unable to machine the fibre at the point of contact. Instead, it exerted compressive force on the workpiece, resulting in the bending of the fibres rather than their shearing. As a result, fibre deflection increased with tool progression, eventually resulting in bending failure occurring, typically below the cutting plane. The interaction between the cutting tool and composite part is modified using a rounded cutting edge [6,13]. When machining composites, sharp cutting edges form a chipping area in front of the tool, whereas rounded edges have pressing and chipping regions. Composites bend and elastically rebound after tool compression, while chipping zone fibres break to form chips. Thrust force increases due to contact and pressure with tool clearance face despite material bounce back. Rake angle influences chip formation, with large positive angles producing continuous chips and small angles generating discontinuous chips. Tool clearance angle does not significantly affect chip formation. There is limited literature exists on the interaction between cutting tool and material at the micro-scale [13]. Most of the existing literature on machining composite materials has focused on tools with positive rake angles, leaving a gap in understanding the chip type produced when using cutters with negative rake angles.

According to Wang and Zhang [6], The degree of damage incurred by CFRP components during machining is significantly affected by both cutting depth and fibre orientation. Decreasing the cutting depth results in less subsurface damage, a finding that is consistent with previous research by Koplev et al. [14]. Bhatnagar et al. [15] conducted a study on the damage incurred by composites during machining, focusing on fibres with orientations ranging from 0° to 90°. The study used depths of cut of 0.1 mm, 0.2 mm, and 0.3 mm, respectively, to characterise the depth of damage. It was found that the depth of damage was lowest for composite materials with fibre orientations in the range of 15° ≤ *θ* ≤ 30°. Above this range, the depth of damage increased gradually up to *θ* = 60°, with a maximum value of 0.5 mm when using a depth of cut of 0.3 mm. For fibre orientations exceeding 60°, the depth of damage increased further with a maximum value of 2.5 mm observed at *θ* = 90° and a depth of cut of 0.3 mm. Composite materials with fibre orientations greater than 90° are prone to significant fibre bending and remain tightly packed in bundles until failure, which can cause rapid failure of the material [16]. This explains the high surface roughness of a machined composite workpiece when machined with the tool of a rake angle of 40° and fibre orientation of 150° [6]. In another study, fibre-matrix debonding and pull-out were investigated and found to increase with larger fibre orientation [17]. In a recent study, li et al. used a proposed FE model supported by experimental validation to investigate damage behaviors of UD-CFRP composites in orthogonal cutting. They found that, multiple pass cutting was recommended due to improved surface integrity and fewer fiber breakages [18].

The available literature on the chip formation mechanism in unidirectional CFRP composites is insufficient, with only a few studies qualitatively analysing certain aspects of the process or modeling it using FEA. Additionally, no research has been conducted on the impact of machining parameters on the quality of CFRP composites during orthogonal cutting, nor on the optimization of process parameters. The study aims to investigate the effect of orthogonal cutting parameters on the surface quality and integrity of unidirectional carbon fibre composites. The research examines the microscale characterization of machined composites, the impact of tools with negative rake angle and rounded cutting edge, and the influence of bounce back on the depth of cut and thrust forces. Response Surface Methodology (RSM) and Analysis of Variance (ANOVA) were used to identify significant machining parameters and develop analytical models to predict machining forces, surface roughness, damage depth, and bounce back. Optimal process parameter values were determined to enhance the quality of machined parts.

## 2. Experimental

### 2.1. Workpiece Material, Tooling, Experimental Setup, and Equipment

**Figure 1:**
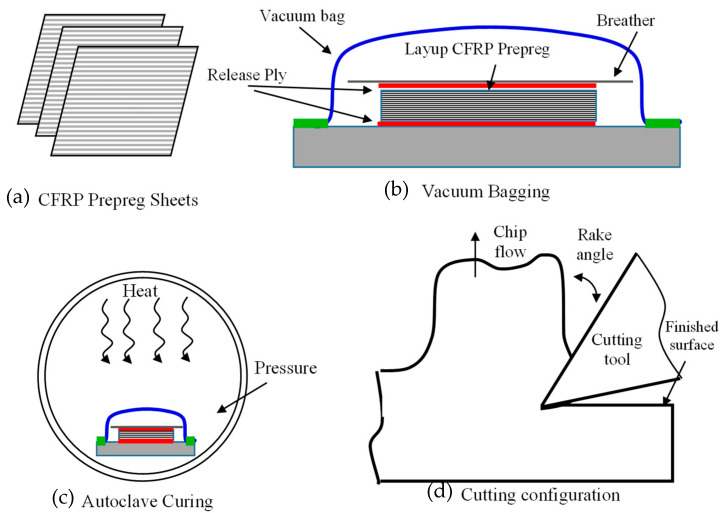
A schematic of the composite preparation and the cutting process (**a**) CFRP Prepeg sheets, (**b**) Vacuum Bagging, (**c**) Autoclave Curing, (**d**) Cutting Configuration.

The CFRP material used in this study was T800S/HexPly^®^ M21 carbon fibre/resin matrix, which was in the form of pre-impregnated sheets with a thickness of 0.26 mm. To prepare the composite material, the T800 pre-impregnated carbon fibre was first cut into the desired dimensions using a sharp cutter. The work surface was cleaned thoroughly to avoid contamination. A plywood sheet was then placed on the processing table and covered with release film, followed by laying the breather fabric and T800 pre-impregnated carbon fiber. The orientation of the fibres was marked using a pen, and the process was repeated until the desired number of layers was achieved. Laminates were fabricated with the fibre orientation set at 0°, 45°, 90°, and 135°, respectively. To prevent resin leakage, sealant tape was applied around the stack’s edges, and the vacuum bagging film was sealed using sealant tape. The vacuum pump was connected to the bagging film to remove air between the layers. The bagged stack was then cured at 130 °C. Once cured, the laminate was allowed to cool down before removing the vacuum bagging film and releasing the film, see Figure 1a–c. The properties of the fibre and matrix phases can be found in Table 1. To perform the cutting, solid tungsten carbide single-point cutting tools were used, which were brazed onto a steel body with a circular shank. The cutting edges were ground to produce tools with three different rake angles (−10°, 10°, and 30°), a constant clearance angle of 10°, and a radius of 20 µm, see Figure 1d.

**Figure 2:**
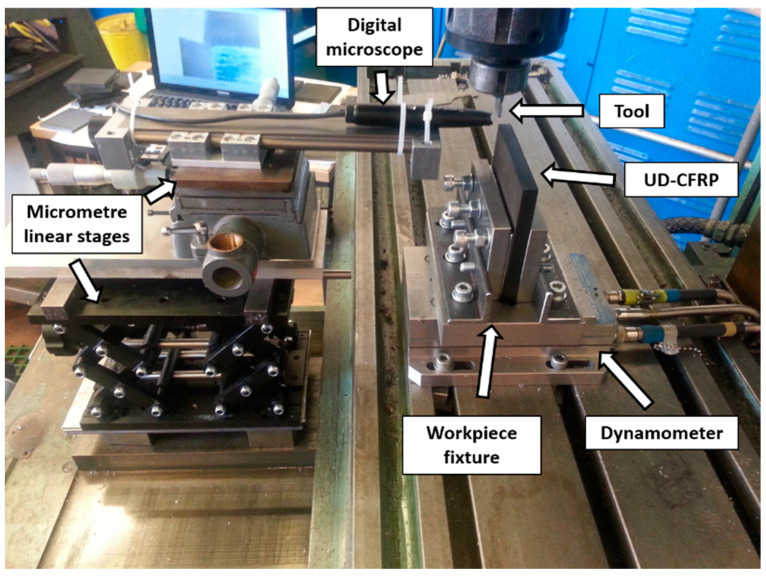
Experimental setup for unidirectional orthogonal cutting.

The experiments for orthogonal cutting were conducted using a manual Cincinnati vertical spindle milling machine. The CFRP workpiece was clamped in a vice and moved along with the worktable while the cutting tool remained stationary in the locked spindle. The setup is illustrated in Figure 2, which is similar to setups used by several other researchers [7,10,21]. To characterise the cutting force and thrust forces, the Kistler 9257A piezoelectric platform dynamometer (Winterthur, Switzerland) was used, connected to Kistler 5011 charge amplifiers, and linked to a data acquisition computer system installed with Dynoware software for signal capture and manipulation. A special fixture was prepared to hold the CFRP workpieces in the optimal fibre orientation in relation to the direction of the cut. The fixture was mounted on the dynamometer and clamped on the machine worktable (Figure 1), similar to the one introduced by Kahwash et al. [22]. Dynamic images of the cutting process were captured at a rate of 15 frames per second using a Supereyes^®^ digital microscope (Shenzhen, China), enabling analysis of chip types, and associated formation mechanisms. However, the acquisition frequency of the unit was only sufficient to capture images from tests performed at the lowest cutting speed level (12 mm/min). The microscope was positioned to view the tool-workpiece interaction side profile, and a precise cutting zone was achieved using three-micrometre linear stages. The digital microscope was additionally outfitted with LED lights linked to a computer running image acquisition software. To make chip formation and damage propagation visible during machining, a thin layer on the surface of the CFRP workpieces was removed using laser ablation to reveal the fibres in the material.

An Alicona Infinite Focus G5 microscope (Graz, Austria) was employed to assess the surface roughness of the workpiece by scanning along its centreline with a lateral resolution of 4 µm and a vertical resolution of 2 µm. It was also used to obtain the topography of the machined workpiece. Based on the topography and the depth of cut, the bounce back was estimated. To characterise the extent and type of damage and defects in the machined workpiece, a scanning electron microscope (SEM) was employed.

### 2.2. Experimental Design and Procedure

The present study evaluated the effect of four variable parameters: fibre orientation (four levels), cutting speed, depth of cut, and rake angle (three levels each), as given in Table 2. Two experimental designs were formulated, with the first being a full factorial experiment involving 36 sets of experiments (N), each performed at three different cut depths (50, 100, and 150 µm), resulting in a total of 108 tests, as shown in Table 3. The responses evaluated included chip type, cutting, and thrust forces, and workpiece surface quality and integrity. Finally, a new tool was used for each test.

The second experimental design implemented in this study involved a face-centred central composite design, which used RSM and focused on a subset of the initial test plan (as shown in Table 3). Three replications of the centre point (experiments 23) were conducted to consider three levels of each variable, except for the fibre orientation of 0°, which was not considered. In addition to the response measures evaluated in the initial full factorial test array, the depth of workpiece subsurface damage, surface roughness, and degree of fibre bounceback were also assessed for the RSM trials. After conducting the experiments, ANOVA was used to identify statistically significant process parameters, and analytical models was developed using Design-Expert 7.0 to predict machining forces, depth of workpiece damage, surface roughness, and bounce back.

### 2.3. Machining Forces

**Figure 3:**
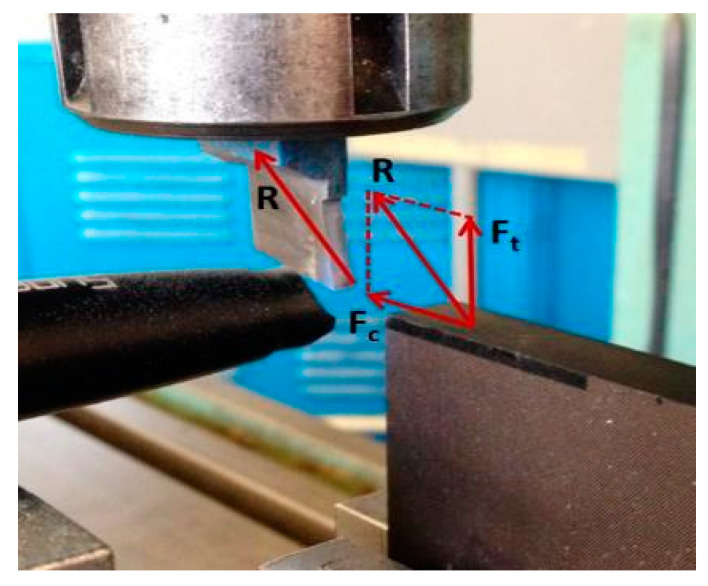
The analysis of resultant machining force.

Figure 3 illustrates a schematic of the cutting and thrust forces that act on the tool during machining. To study the impact of cutting parameters on these forces, the calculated values were determined when machining samples with cutting depths of 200 µm and 250 µm.

## 3. Results and Discussion

### 3.1. Chip Type and Morphology

Composite materials can experience three types of failure modes. Wang et al. [5] have identified five failure modes that can occur in composite materials. Type I failure is localized at the interface of the fibre and matrix, whereas Mode II failure results in fractures perpendicular to the fibre orientation under bending stresses. In Mode III failure, the failure occurs due to compression-induced shear stresses across the fibre axis. Mode IV failure involves a combination of interfacial shearing and compression-induced shear stresses across the fibre axis. Lastly, Mode V failure primarily occurs due to compressive loads perpendicular to the fibres.

**Figure 4:**
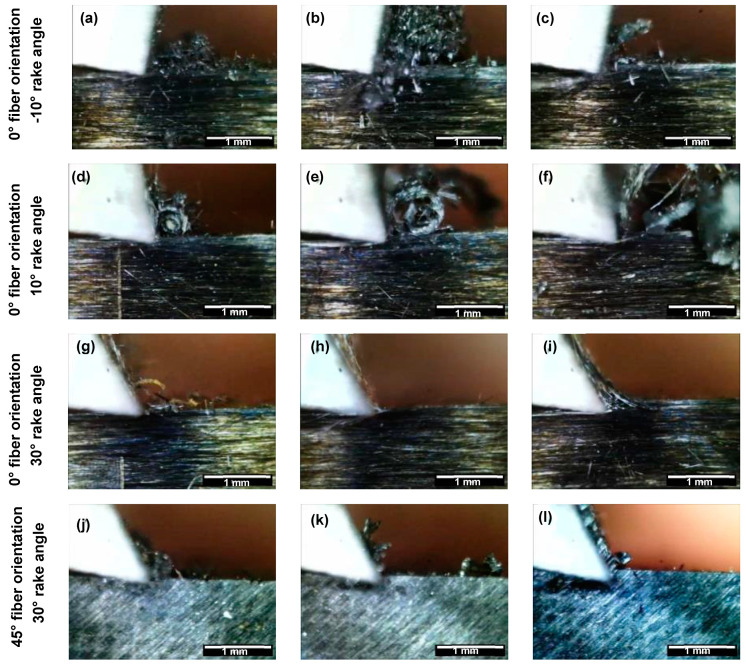
Chip formation at a depth of cut of (**a**,**d**,**g**,**j**) 50 µm, (**b**,**e**,**h**,**k**) 100 µm and (**c**,**f**,**i**,**l**) 150 µm. (The white scale bar = 1mm).

The study utilized a digital microscope camera to capture photos, which were analysed to investigate the relationship between tool rake angle, depth of cut, and fiber orientation on composite chip formation mechanisms during cutting. The results revealed that cutting at depths of 50 µm and 100 µm with a negative rake angle tool produced powdery chips, as depicted in Figure 4a and Figure 4b, respectively. The chips exhibited discontinuities when the depth of cut was increased to 150 µm, as shown in Figure 4c. On the other hand, a tool with a small positive rake angle of 10° produced continuous curling chips with increasing thickness and depth, as shown in Figure 4d–f.

The morphology of chips observed during orthogonal cutting provides valuable information about the underlying mechanism of chip formation. Negative rake angles trigger the Type II mechanism, where fibre buckling leads to fracture due to the sheer force of the tool, resulting in powdery chips. On the other hand, low positive rake angles cause the fibre-matrix interface to fail and the fibres to bend, leading to the continuous formation of chips. Even with a larger rake angle of 30°, chips remained continuous, sliding along the tool’s rake face. Similarly, the chip morphology observed with a 30° rake angle tool showed increased thickness and cutting depth, as seen in Figure 4g–i. At a fibre orientation of *θ* = 45°, the chip morphology showed a significant change as the depth of cut increased for all tool rake angles, as depicted in Figure 4j–l. A shift from powdery chips to continuous chips was observed with increasing depth of cut. This change in chip morphology was due to the development of chips becoming independent of the tool rake angle when cutting at a fibre orientation of *θ* = 45°. The results of our analysis suggest that controlling the fibre orientation and the rake angle of the tool can significantly affect the chip morphology and ultimately impact the quality of the machined surface.

**Figure 5:**
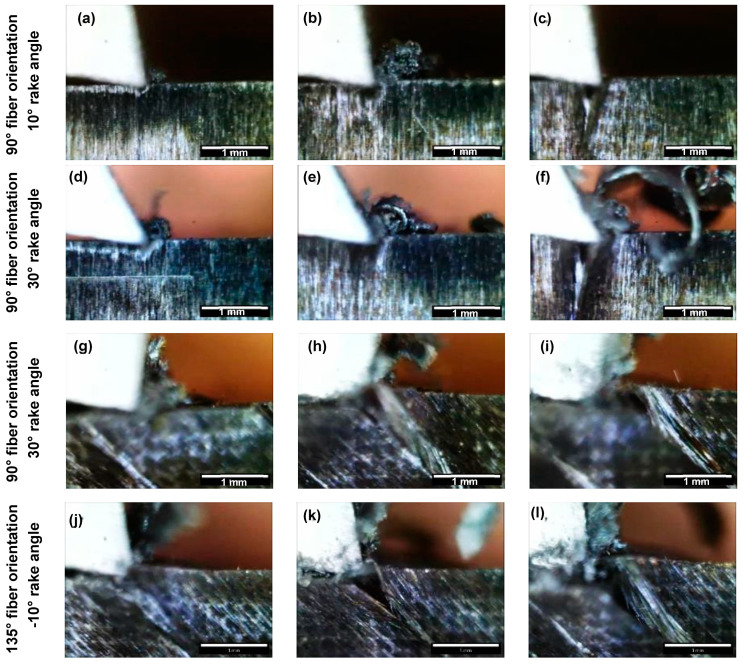
Chip formation at a depth of cut of (**a**,**d**,**g**,**j**) 50 µm, (**b**,**e**,**h**,**k**) 100 µm and (**c**,**f**,**i**,**l**) 150 µm. (The white scale bar = 1mm).

It was observed that during machining with tools having negative or small positive rake angles and a fiber orientation of *θ* = 90°, powder-like chips were formed at cutting depths of 50 µm and 100 µm, but negligible material removal/chip formation was visible at a cutting depth of 150 µm, as shown in Figure 5a–c. However, increasing the cutting depth to 150 µm resulted in negligible material removal/chip formation, except for a few small splinters. This phenomenon was attributed to the rounded cutting edge’s inability to effectively shear the fibres, resulting in fiber bending instead of fracture. Furthermore, the bent fibres undergo elastic recovery after the tool passes over the machined surface, leading to negligible material removal as reported in [12]. Figure 5d–f illustrates the change in chip morphology when using tools with a rake angle of α = 30°. The authors observed the formation of irregularly curled chips with increasing thickness at higher cutting depths. This was due to a decrease in the contact area between the tool and workpiece, leading to greater stress concentration near the contact area and fiber fracture. The observed chip morphology under positive rake angles was found to be consistent with previous studies in the literature. The observed chip type under positive rake angles was found to be consistent with the previous literature [23].

**Figure 6:**
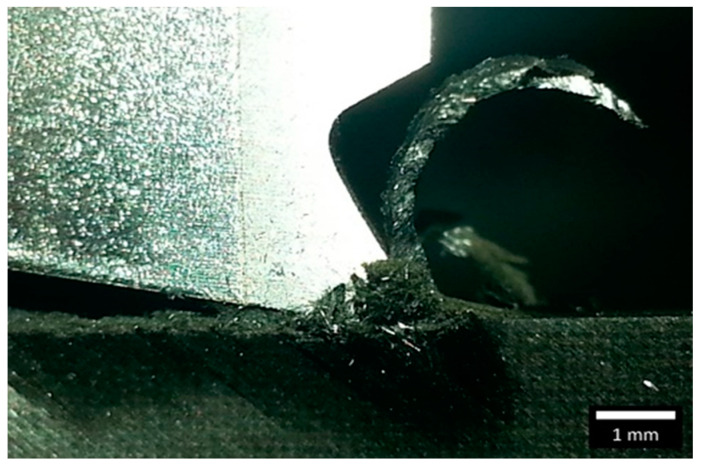
Chip formation for experiment 34 at fibre orientation of 135°, rake angle of 30°, and depth of cut of 150 µm. (The white scale bar = 1 mm).

Li et al. [23] reported consistent chip morphologies when machining composites with fibre orientations ranging from 75° to 180°. However, our study observed a change in chip morphology when machining composites with fibre orientations between 90° and 135°. The use of a negative rake angle tool, Figure 5g–i, resulted in brittle, discontinuous chips mixed with resin powder, whereas the discontinuity was slightly improved with the use of low positive rake angles as shown in Figure 5j–l. Moreover, to investigate the impact of positive rake angles, tools with a higher rake angle of 30 degrees were used. Tools with a rake angle of α = 30° produced thick, continuous chips at each cutting depth (Figure 6), which were captured at the end of the cut using a conventional camera.

### 3.2. Chip Formation Mechanisms

**Figure 7:**
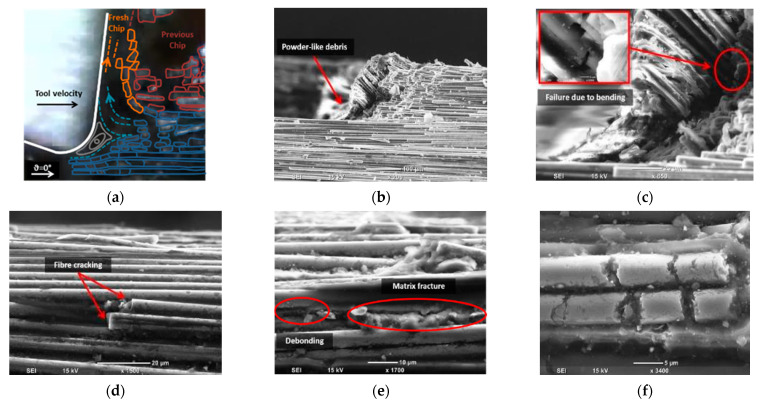
Chip formation for samples with a fibre orientation of 0° and a rake angle of −10°: (**a**) a schematic diagram overlaid on a digital image, (**b**) the chip root, (**c**) a magnified SEM image of the chip root, (**d**) a side view showing fibre damage, (**e**) a side view SEM image showing matrix damage, and (**f**) a top view SEM image of the machined surface.

To understand the material removal and chip formation for each test condition, digital microscope images taken during cutting were used in conjunction with high magnification SEM micrographs to enhance understanding of the observed behaviour. These images were utilised to develop a schematic representation of the material removal process. However, only the samples machined at the lowest cutting speed were analysed to mitigate the effects of tool deceleration. When machining CFRP with a fibre orientation of *θ* = 0° using negative rake angle tools, Type II chip formation was observed, with fibre buckling identified as the primary failure mechanism at the tip of the cutting tool [13]. However, a closer examination of the images revealed a complex chip formation process attributable to the tool’s round cutting edge, as depicted in the schematic shown in Figure 7a. During the cutting of the samples, the composite material flow split into two sections in front of the tool, and the accumulation of chips as resin powder and short fibres was observed at the tip of the cutting tool.

Figure 7b,c illustrate powder-like debris remains at the chip root after the composite material is cut. During cutting, the tool pushes the fragmented material forward, causing the material above it to lift and bend fibres until they fail near the cutting tool tip (as shown in Figure 7c). Fibre failure occurs primarily due to cracking or breakage, which propagates orthogonally to the fiber direction (Figure 7d). Additionally, Figure 7e highlights matrix fracture and debonding that occurs between two adjacent fibres. The composite material flowing under the cutting tool exhibits fractured fibres due to the downward thrust force applied, as depicted in Figure 7f. These findings are consistent with previous work by Wang and Zhang [6].

**Figure 8:**
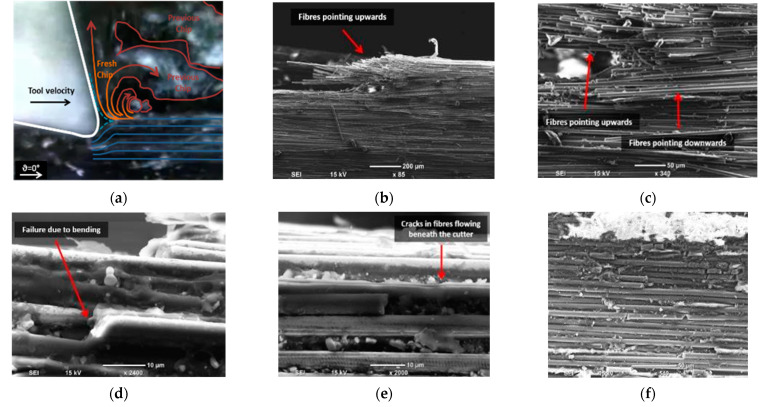
Chip formation at fibre orientation 0° and tool rake angle 10°, (**a**) a schematic overlaid on a digital image; (**b**) The chip root; (**c**) a magnified view of the chip root; (**d**) a side view showing fibre failure; (**e**) a side view revealing fibre crack; and (**f**) a top view of the machined surface.

**Figure 9:**
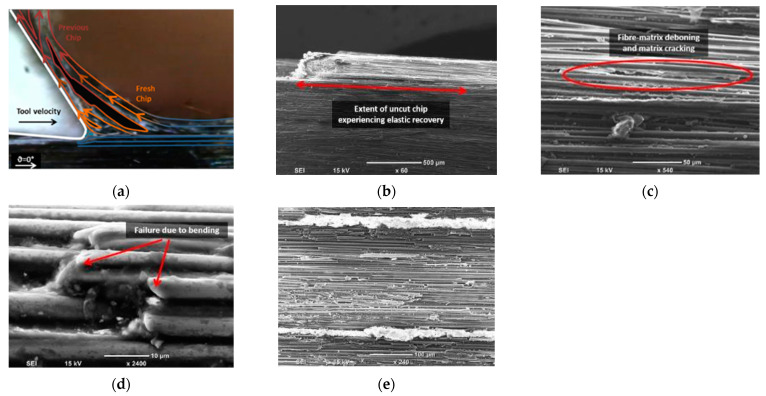
Chip formation observed at fibre orientation 0° and tool rake angle 30°: (**a**) schematic overlaid on the digital image; (**b**) The chip root; (**c**) side view highlighting matrix damage; (**d**) side view showing fibre failure; (**e**) top view of the machined surface.

**Figure 10:**
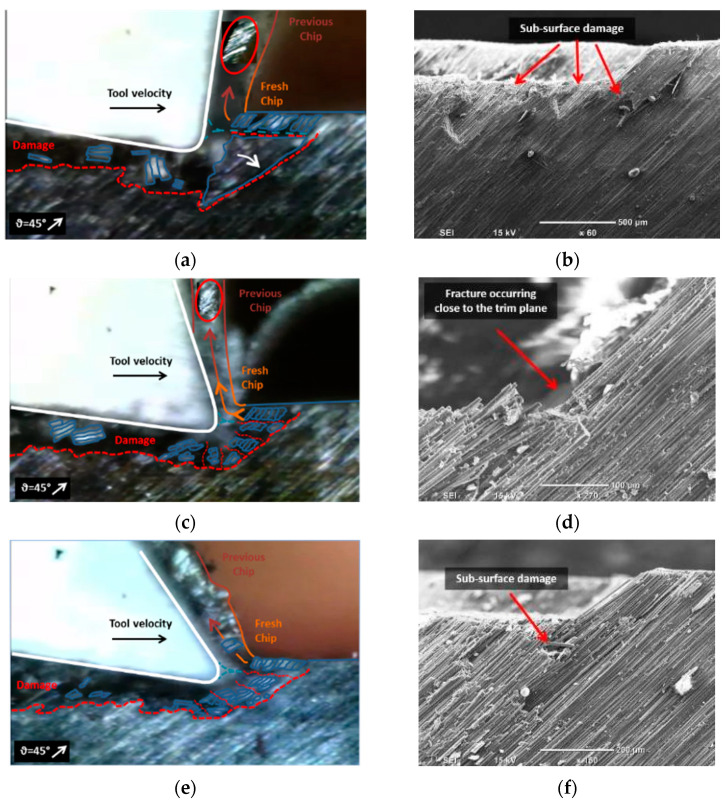
Chip formation at fibre orientation 45° and tool rake angle (**a**,**b**) −10°; (**c**,**d**) 10°; (**e**,**f**) 30°.

In machining experiments using tools with positive rake angles, the mechanism of chip formation and material removal led to the formation of continuous chips. Corresponding schematics can be found in Figure 8a and Figure 9a. Similar to the previous machining condition, the tool separated the flow of the material into two sections, see Figure 8b,c. At the chip root, uncut deformed fibres were visible, pointing downwards and upwards. Due to the rounded shape of the cutting edge, a fibre buckling zone was always present, but its size was reduced compared to machining using tools with a negative rake angle. When the cutting tool was removed, the composite workpiece exhibited elastic recovery behaviour, causing the chip to return to its horizontal position. Fibre bending failure was evident, and cracks propagated orthogonally to the fibre axis, as illustrated in Figure 8d. This pattern of crack propagation is a distinctive characteristic of a Type I mechanism, as noted in [13]. The cutting tool exerted a downward force, which caused the deformation of the composite fibres. This deformation resulted in the formation of cracks that propagated across the fibres and allowed the material to deflect below the tool, as depicted in Figure 8e. The top view of the machined workpiece is depicted in Figure 8f, which shows multiple cracks resulting from the pressure exerted by the tool during cutting. In all cases, fibre failure occurred very close to the cutting tool tip when the rake angle was 10°, regardless of the depth of cut. This was because the cutting tool with a small rake angle led to a rapid bending deformation of fibres. When a higher rake angle (α = 30°) was used, the material sliding against the tool face with a higher rake angle resulted in chips being created from two distinct areas in front of the cutting tool (Figure 9), as noted by Li et al. [23]. Fibres underwent more gradual bending at the tool tip than in trials with a low positive rake angle, with evidence of additional workpiece separation originating further away from the cutting edge. This was due to plying peeling (delamination), which became more significant and initiated farther ahead of the tool with increasing cut depth, as Zitoune et al. reported [10]. The voids present in the workpiece were due to fibre-matrix debonding and cracking of the matrix, as highlighted in Figure 10c, with fracture caused by progressive bending perpendicular to the fibre axis (Figure 10d). Figure 9b shows that the uncut chip underwent elastic recovery following the removal of the tool. The top-down SEM view shows multi-fractured fibres, similar to the previous machining conditions (Figure 9e–f).

Workpieces with fibre orientation of *θ* = 45° exhibited similar chip formation mechanisms regardless of the rake angles employed. As the tool advanced, it caused the fibres to experience multi-fractures perpendicular to the fibre axis in front of the cutting tool (see Figure 10). The first fracture occurred close to the trim plane. Due to the deeper fractures, the fibres fractured above the cutting tool formed the chip, while those below flowed underneath along with other damaged material. High magnification SEM images provided evidence for the cracks’ propagation normal to the fibre axis and the existence of subsurface damage. Analysis of the chip’s fibre direction revealed that the composite workpiece underwent shearing along the fibre axis, especially close to the tooltip. Additionally, the shearing increased as the tool rake angle decreased. This forming mechanism is consistent with Type III, where interlaminar scissoring shear caused the failure.

**Figure 11:**
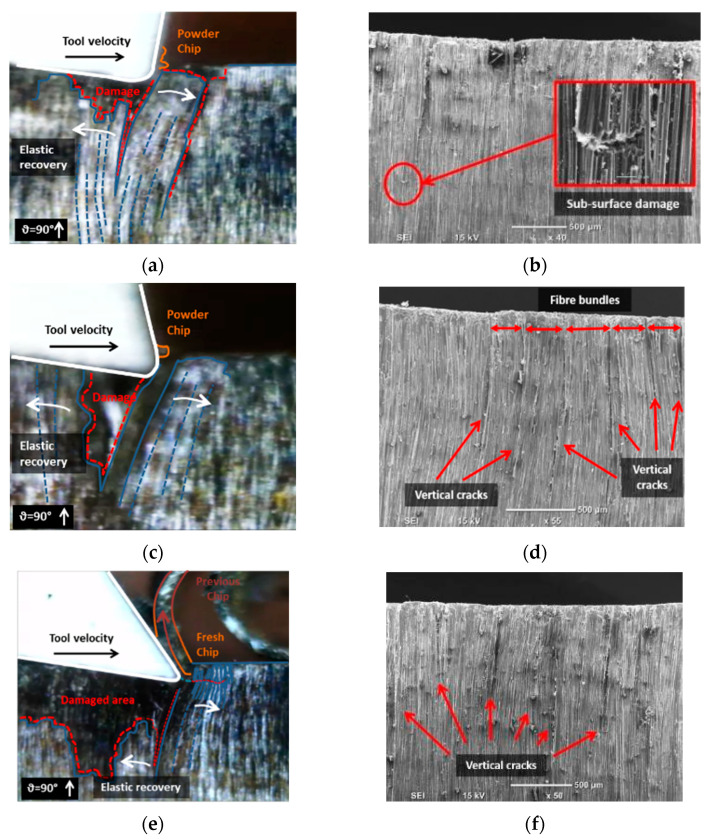
Chip formation at fibre orientation 90° and tool rake angle (**a**,**b**) −10°; (**c**,**d**) 10°; (**e**,**f**) 30°.

For fibre orientation of 90°, comparable performance was shown at tool rake angles of −10° and 10° (Figure 10). The formation mechanism of chips was similar to that reported in Pwu et al. research [12]. Progression of the cutting tool caused significant bending deformation on the CFRP fibres, resulting in considerable subsurface damage owing to delamination of the deformed composite material. Concurrently, fibre compression near the tool tip resulted in failure and the release of powder-like chips. However, elastic recovery caused the bent fibres to return to their initial position after the tool had passed. The combined effect of tool pressure and elastic recovery led to an increase in the material separation depth. The observation of fibre bundles between two consecutive vertical cracks, indicated by the red arrows in Figure 11, confirms the reported behaviour of material separation. When using tools with a rake angle of 30°, continuous chips were formed. As the tool advanced, the fibres experienced bending deflection. However, the high positive rake angle of the tool decreased the cutting contact area, leading to the formation and horizontal propagation of a crack ahead of the tool tip. The material above the crack flowed upward along the rake face of the tool, resulting in the formation of a chip. As for samples with a fiber orientation of 45°, the composite material was sheared in the direction parallel to the orientation of the fibres. As a result, the region beneath the crack displayed elastic rebound and brushed against the clearance face. Additionally, fiber failure caused by bending was observed below the cutting plane (Figure 11b), and cracks propagated perpendicular to the fiber axis regardless of the rake angle.

**Figure 12:**
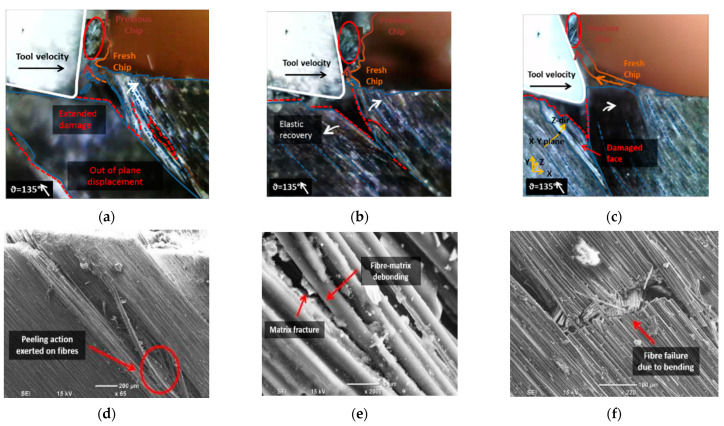
Chip formation at fibre orientation 135° and tool rake angle of (**a**) −10°; (**b**) 10°; (**c**) 30°; and (**d**–**f**) workpiece damage on the side view.

When cutting CFRP with a fibre orientation of *θ* = 135°, chip formation across the different rake angles (Figure 12) was largely alike, identifying a Type I mechanism. The tool engages with the fibres during cutting, causing the material to be lifted and peeled. Fibres that undergo significant deformations fail, forming cracks propagating orthogonally to the fibre axis directed on the top workpiece surface, as shown in Figure 12f. As previously mentioned, for samples with a fibre orientation of *θ* = 90°, the damage depth was amplified due to the combined effect of cutting force and elastic recovery of the workpiece, resulting in material separation. The separation is attributed to matrix fracture and fiber-matrix debonding between adjacent fibres, as observed in Figure 12d. The deformation of the carbon fibres in this area indicates material separation resulting from fiber peeling, with a magnified view presented in Figure 11e and a side view in Figure 12f.

### 3.3. Analysis of Response Surface Methodology Experimental Design

The experimental results of the RSM design, with the input parameters outlined in Table 3, are presented in Table 4. An analysis of variance (ANOVA) was conducted to determine the significant parameters that affected each output measure. The analysis was performed with a confidence level of 95%. Table 5 summarises the p-values calculated from the ANOVA for each parameter xi and the corresponding quadratic terms *x*_2*i*_, and their interactions *x_i_ x_j_*, in relation to the thrust force, cutting force, damage depth, workpiece roughness, and bounce back. The models that describe each of the output responses and their corresponding coefficient of determination (R-squared) values are presented in Table 6. The results indicate that the 2-factorial interaction provides good predictions for the thrust force, cutting force, and damage depth, while a quadratic model provided a better fit for the roughness and bounce back. The R-squared values for all responses ranged from 0.87 to 0.98, demonstrating a strong correlation between the model and measured data.

The general form of the model to predict the different output variables (*var*) is expressed in Equation (1):

(1) $$var=a+bx_{1}+cx_{2}+dx_{3}+ex_{4}+fx_{1}x_{2}+gx_{1}x_{3}+hx_{1}x_{4}+ix_{2}x_{3}+mx_{2}x_{4}+nx_{3}x_{4}+px_{1}^{2}+qx_{2}^{2}+rx_{3}^{2}+sx_{4}^{2}$$

where *x*_1*:*4_ is the orientation of the fibres, tool rake angle, cutting depth, and speed. The coefficients of the model for each of the output factors were determined using Design-expert software V7.0 and are listed in Table 7.

### 3.4. Workpiece Damage and Surface Integrity

**Figure 13:**
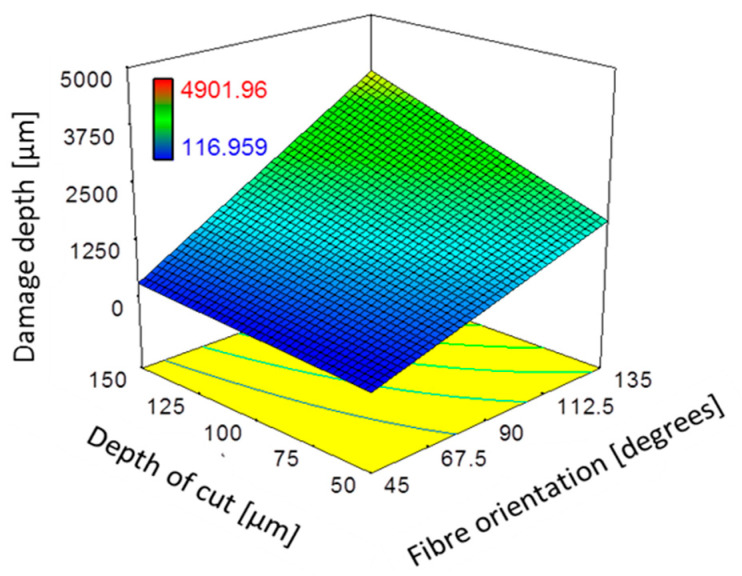
The combined effect of depth of cut and fibre orientation and on damage depth for 10° and $V_{c}=$ 570 mm/min.

**Figure 14:**
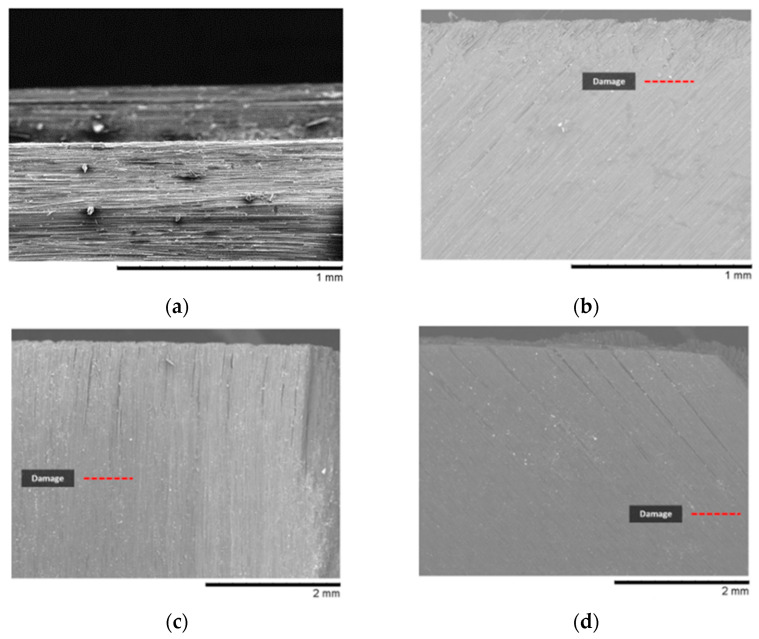
Damage in machined workpiece for depth of cut 50 µm and fibre orientation (**a**) *θ* = 0°, (**b**) *θ* = 45°, (**c**) *θ* = 90°, and (**d**) *θ* = 135°, which correspond to experiments 5, 14, 23, and 32 in Table 3, respectively.

The ANOVA results revealed that fibre orientation, depth of cut, and the corresponding interaction were statistically significant factors (*p*-value < 0.05) affecting the depth of workpiece damage, which agrees with the literature [6], as shown in Table 5. The model graph presented in Figure 13 provides further insight into the impact of the aforementioned parameters on damage depth. By fixing the unshown variables on the axis to the mid-level stated in Table 2, the effect of fibre orientation and depth of cut on damage depth can be observed. It is evident from the graph that damage depth increases significantly with greater fibre orientation and cutting depth, consistent with findings in the existing literature [6,14,15,17]. The combination of these effects resulted in significant damage, as demonstrated by the SEM micrographs in Figure 14, which depict the variation in workpiece damage with respect to fibre orientation. The images clearly show that as the fibre angle increases, the depth of damage also increases, with the highest level observed when both effects were present. In contrast, a small damage depth was observed for fibre orientation of 0°, where the damage depth was primarily limited to the vicinity of the trim plane, consistent with previous findings reported in the literature [14,24].

**Figure 15:**
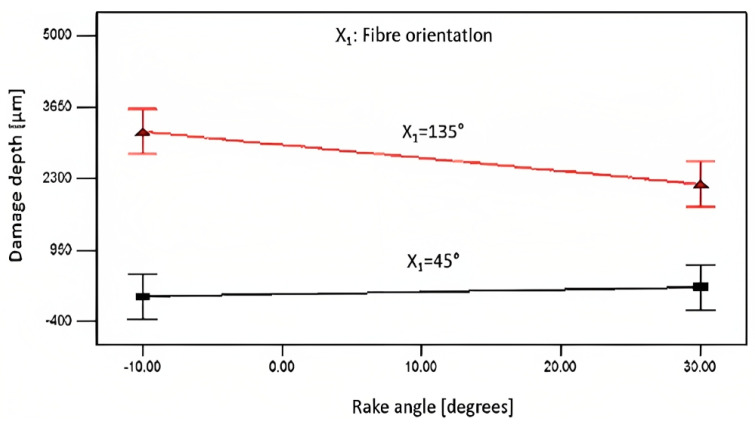
Effect of fibre orientation and tool rake angle on the depth of cut of 100 µm and $V_{c}=570$ mm/min.

The influence of tool rake angle on damage depth varied with the workpiece at different fibre orientations, as shown in Figure 15 for *θ* = 45° and *θ* = 135°. For CFRP, at a fibre orientation of 135°, the depth of damage was significant when using negative or low positive rake angle tools, which was dominated by material deformation instead of shearing. This led to a lower-than-expected depth of material removed with significant protruded/uncut fibres on the machined surface. On the other hand, a higher quality surface was achieved by using a positive tool rake angle such as 30°, resulting in a thicker chip and relatively shallower damage depth, even though the cutting depth surpassed the intended value.

**Figure 16:**
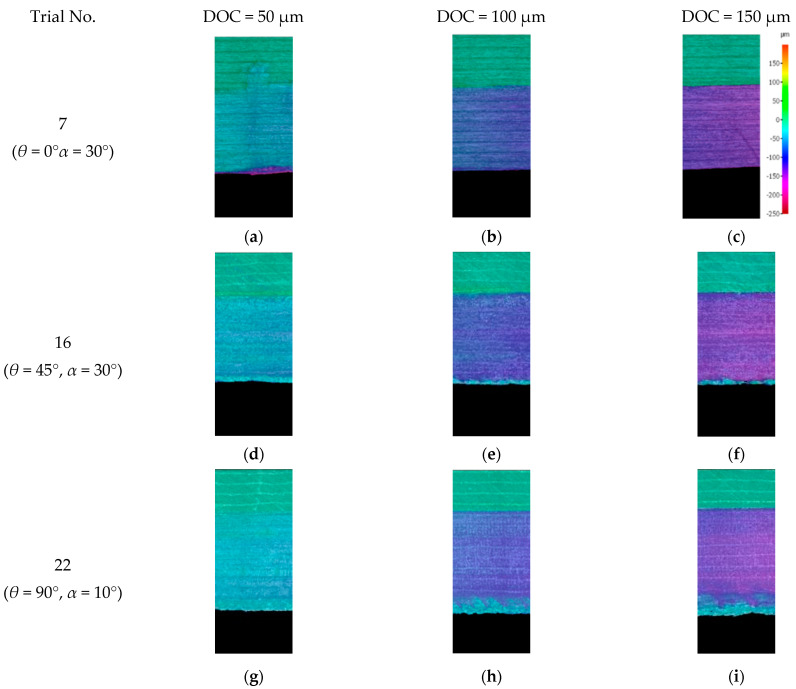
The measured depth of cut from scanned surface profile for (**a**–**c**) Experiment 7 (orientation angle = 0° rake angle = 30°); (**d**–**f**) Experiment 16 (*θ* = 45°, *α* = 30°); and (**g**–**i**) Experiment 22 (orientation angle = 90°, rake angle = 10°).

**Figure 17:**
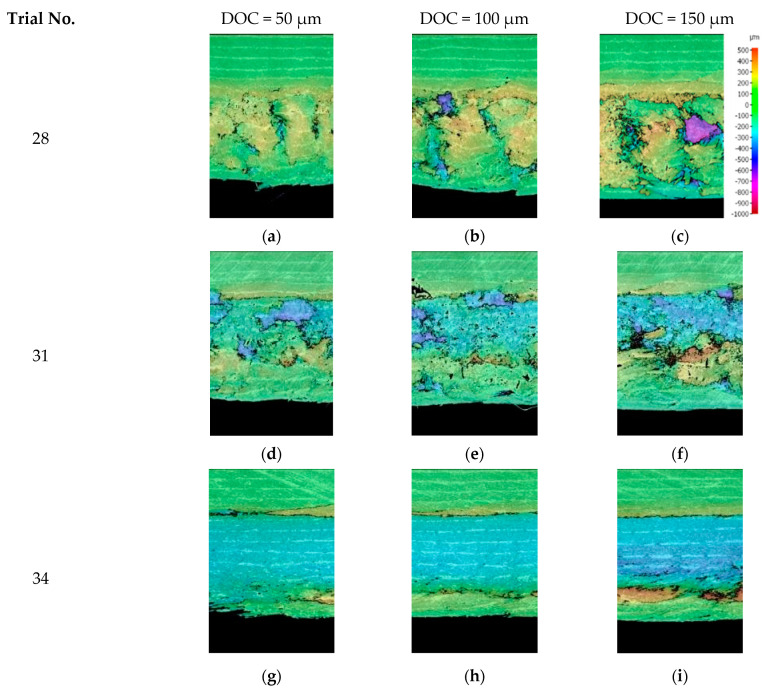
The measured depth of cut from scanned surface profile for fibre orientation of 135° and cutting speed of 12 mm/m at different rake angles of (**a**–**c**) −10°; (**d**–**f**) 10°; and (**g**–**i**) 30°.

The scanning images obtained from the machined surface in Figure 16 and Figure 17 revealed the occurrence of out-of-plane deformation during cutting, which increased as the cutting depth and the fibre orientation increased. This effect was particularly noticeable when machining at a fibre orientation of 135°, resulting in significant out-of-plane displacement, as depicted in Figure 17. Moreover, due to the extensive elastic recovery that occurred during machining at fibre orientations of 90° (Figure 11) and 135° (Figure 12), evaluating the damage depth was challenging because the damaged areas often re-closed.

**Figure 18:**
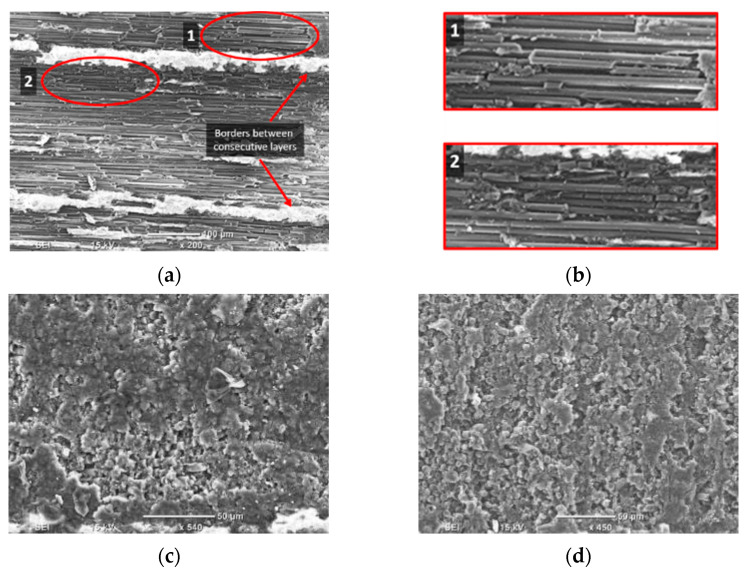
SEM images of the machined surface for (**a**) Experiment 4 (*θ* = 0°); (**b**) magnification of Experiment 4; (**c**) Experiment 13 (*θ* = 45°); and (**d**) Experiment 22 (*θ* = 90°). The machining direction is from left to right.

**Figure 19:**
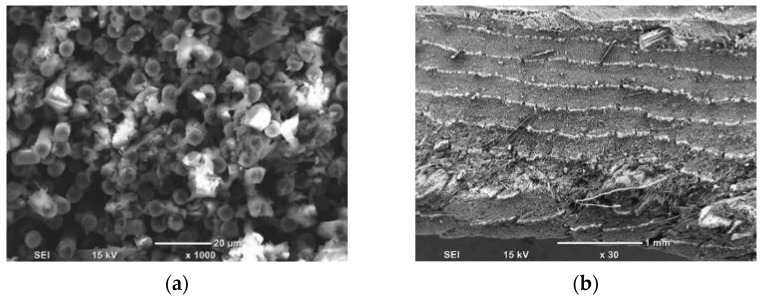
SEM images of machined surfaces for (**a**) Experiment 31 (*θ* = 135°, α = 10°); and (**b**) Experiment 34 (*θ* = 135°, α = 30°), with machining direction from left to right.

High magnification SEM analysis showed that the machined surfaces for workpieces with a fibre angle of *θ* = 0° appeared similar irrespective of tool rake angle, with exposed uncut fibres and some matrix debris prevalent (Figure 18a). The main types of damage observed during cutting were fibre fracture and matrix crushing, as shown in Figure 18b. These were caused by the cutting force on the material. Workpieces with a fibre orientation of 45° (Figure 19c) and *θ* = 90° (Figure 18d) exhibited similar surface quality, with areas covered with the matrix, unlike those with a fibre orientation of 0°. Additionally, fractured fibres were visible on the surface of the machined workpiece. Similar SEM results have been reported in the literature when machining CFRP with fibre orientations of 0° [10,21] and 90° [9,10,21]. As for material with a fibre orientation of 135° (Figure 18), exposed fibres and fractured matrix resin on the surface were observed in Figure 19a. Borders marks between consecutive layers were clearly seen in Figure 19b, where out-of-plane displacements due to machining were visible.

### 3.5. Surface Roughness

**Figure 20:**
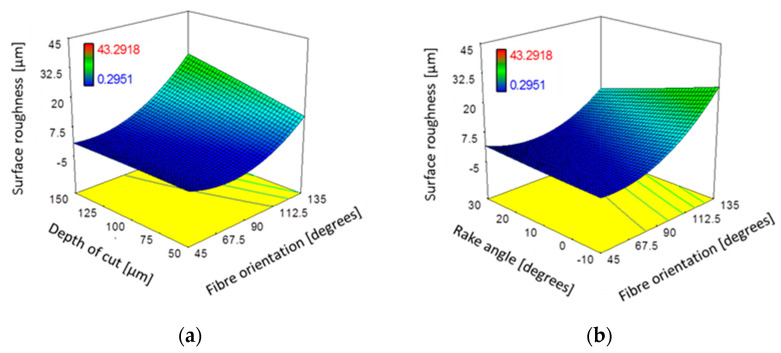
The combined effect on surface roughness (*R*_a_) of (**a**) depth of cut and fibre orientation at constant α: 10°; *V*_c_: 57 mm/min; and (**b**) fibre orientation and tool rake angle at constant DOC: 100 µm; *V*_c_: 570 mm/min.

Table 5 shows that workpiece surface roughness was significantly affected by fibre orientation, cutting depth, and tool rake angle, and a significant interaction between the cutting depth and tool rake angle for samples with different fibre orientations. Figure 20 illustrates the response surface plots that display the impact of the essential process parameters on surface roughness. A higher workpiece fibre orientation generally resulted in increased surface roughness levels, which can be attributed to the chip formation mechanism discussed earlier. The depth of cut had a particularly significant effect on samples with a fibre orientation of *θ* = 135°, resulting in an increase in surface roughness due to the large material removal that was bent and compressed by the tool. In contrast, lower surface roughness occurred when using tools with a high positive rake angle, which was clearly distinct at a fibre orientation of *θ* = 135°.

**Figure 21:**
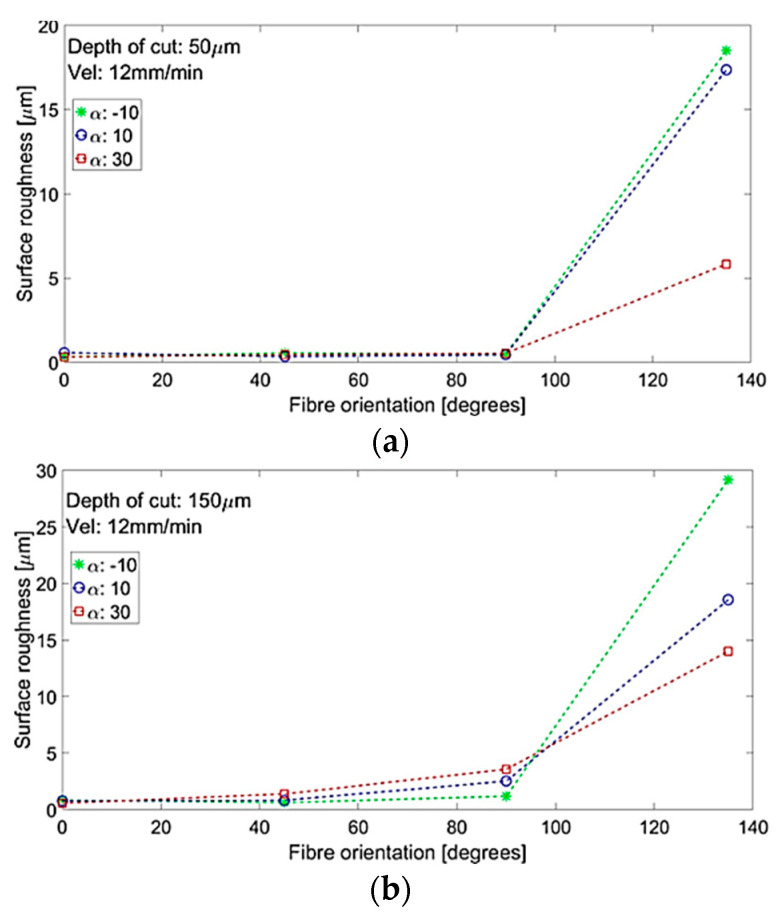
Surface roughness and fibre orientation at various tool rake angles at depths of cut of (**a**) 50 µm and (**b**) 150 µm.

Similar trends in surface roughness with respect to fibre orientation are demonstrated in Figure 21 when using different rake angles tools at cutting depths of 50 µm and 150 µm. The level of surface roughness was relatively low (less than 3.5 µm) for fibre orientations up to *θ* = 90°, regardless of the tool rake angle. The obtained results were consistent with those reported by Wang and Zhang [6], although a slight increase in surface roughness was noticed in the present study when machining samples with a cutting depth of 150 µm (from 2 µm to 3.5 µm) as the fibre orientation varied from 45° to 90° [25]. A significant increase in surface roughness was observed at larger fibre angles (*θ* = 135°) for both depths of cut. The surface quality was found to be dependent on the rake angle, with a lower surface roughness achieved when increasing the tool rake angle. The advantages of using a cutting tool with a large positive rake angle are evident in Figure 17g–i, where samples with improved surface quality were observed for a rake angle of 30°. On the other hand, using cutting tools with a negative or a low positive rake angle to composite machine materials can lead to unsatisfactory surface quality and significant deformation and damage to the surface being machined, as demonstrated in Figure 17a–f.

### 3.6. Bounce Back

**Figure 22:**
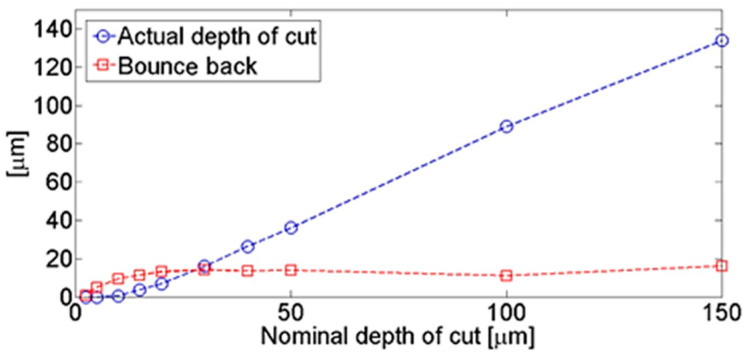
Bounce back and actual cutting depth for a fibre orientation of 0°, a rake angle of −10°, and a cutting speed of 12 mm/min (Experiment 1).

The bounceback effect was conducted by examining the surface profile of the machined samples at different depths of cut. The surface profile was obtained by scanning the machined surfaces using the Alicona Infinite Focus G5 optical microscope and constructing the profiles at the centre of the cut. As shown in Figure 16 and Figure 17, the bounceback variation with respect to the depth of cut is illustrated in Figure 22, which is in agreement with Wang and Zhang [6]. For small nominal depths of cut, the material exhibited significant bounce back even without cutting. This behaviour continued until the nominal cutting depth was approximately half the radius of the cutting tool edge, i.e., 10 µm. Increasing the nominal cut depth beyond this point led to an increased actual cutting of the workpiece, with the amount of bounceback continuing to increase but at a lower rate. At a nominal depth of cut of around 20 µm, the level of bounceback started to plateau and remained constant thereafter, with further increases in the nominal depth of cut resulting in an actual increase in the depth of cut.

**Figure 23:**
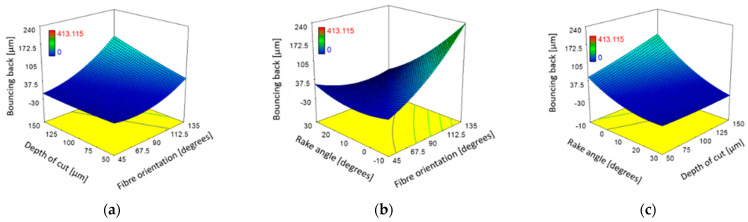
The combined effect on bounce back for (**a**) fibre orientation and cutting depth at constant α: 10°; *V*_c_: 570 mm/min; (**b**) fibre orientation and rake angle at constant DOC: 100 µm; *V*_c_: 570 mm/min; (**c**) rake angle and cutting depth of constant *θ*: 90°; *V*_c_: 570 mm/min.

Table 5 indicated that tool rake angle, fibre orientation, and their interaction have statistically significant effects on the level of bounce back during machining. On the other hand, the depth of cut and its interactions with fibre orientation and the tool rake angle were marginally below the 95% significance level. They were still considered important for further investigation. Figure 23 shows the influence of these factors on fibre bounce back through response surface plots. Increasing fibre orientation generally resulted in greater bounce back [6]. The bounce back was also worsened by increasing the depth of the cut and decreasing the tool rake angle. Figure 23b illustrates the influence of rake angle and fibre orientation on the bounce back and can be further explained by referring to Figure 23. The cases that resulted in the minimum and maximum bounce back were analysed. When machining CFRP with a large fibre orientation (*θ* = 135°) using low positive or negative rake angle tools, a significant workpiece deformation occurred instead of cutting. The tool bent and pressed a considerable amount of material, leading to increased bounce back. In contrast, no bounce back was observed when cutting the fibre orientations CFRP samples (*θ* = 135°) using the tool with a large rake angle (α = 30°). In this experiment, the actual cutting depth was greater than the applied nominal cutting depth.

### 3.7. Machining Forces

#### 3.7.1. Cutting Force

**Figure 24:**
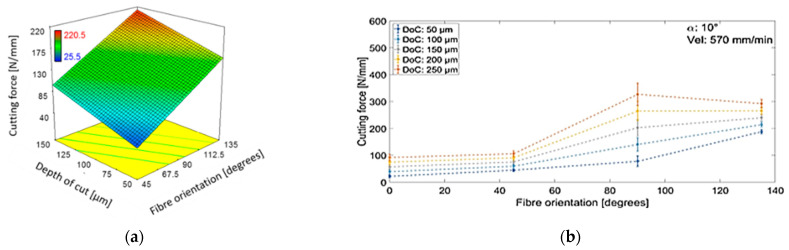
(**a**) Effect of orientation of fibres and depth of cut on the cutting force, and (**b**) the measured cutting force versus fibre orientations at various cutting depths when machining at a rake angle of 10° and cutting speed of 570 mm/min.

Table 5 indicates that composite fibre orientation and depth of cut during machining were significant factors affecting cutting force, as demonstrated by the corresponding response surface plot in Figure 24a. An increase in the fibre orientation of samples or depth of cut resulted in an increase in cutting force. No significant effects were found for quadratic factors or interactions among the variables (see Table 5). The effect of sample fibre orientation on the machining cutting force at different depths of cut is presented in Figure 24b. The results were obtained under a tool rake angle of 10° and a cutting speed of 570 mm/min, a typical condition observed in the experiments. Cutting forces were observed to gradually increase as the workpiece fibre orientation increased from *θ* = 0° to *θ* = 45°, which is consistent with the previously published literature [5,6,7]. The increase in cutting force levels was primarily due to a larger volume of material pressing against the tool [23]. Applying a higher depth of cut caused the curvature of the trend lines for samples with fibre orientation ranging from 45° to 135° to change from positive to negative values. The effect of cutting depth was most significant for workpieces with a fibre orientation of 90°, as this resulted from a large variation in cutting forces. For all the analysed experiments, cutting force was the lowest when machining CFRP with a fibre orientation of 0°.

#### 3.7.2. Thrust Force

**Figure 25:**
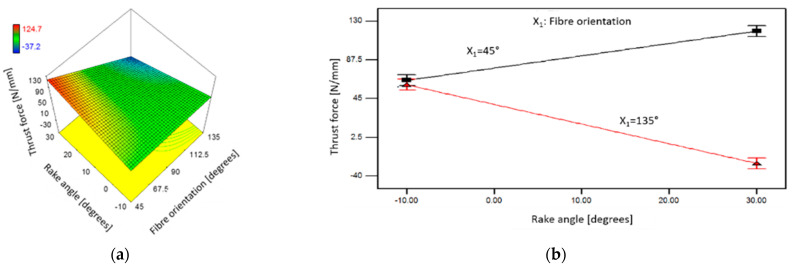
The combined effect of orientation of fibres and tool rake angle on the thrust force when machining at DOC: 100 µm (**a**) 3D view, and (**b**) fibre orientations of 45° and 135°.

**Figure 26:**
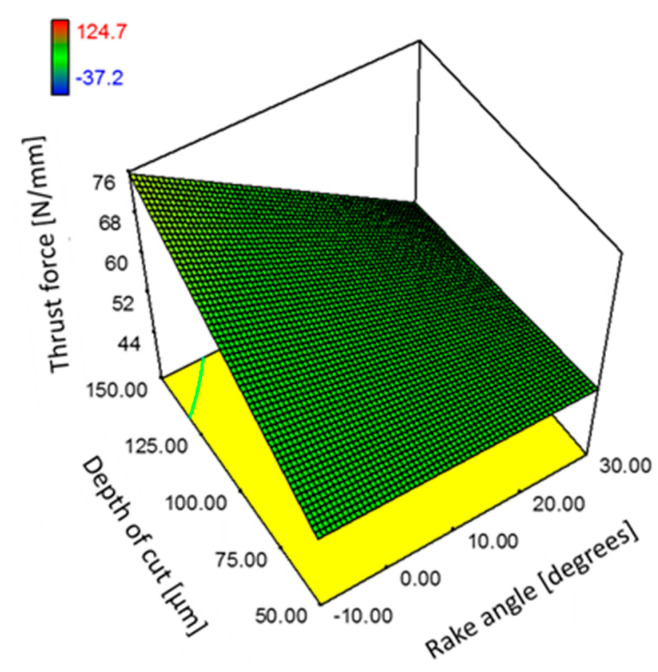
The combined effect of depth of cut and tool rake angle on the thrust force for samples with fibre orientation 90°.

Based on Table 5, the ANOVA results confirm that fibre orientation, rake angle, and depth of cut are significant factors for the thrust force. Figure 25 and Figure 26 illustrate the corresponding response surface plots that show the impact of these parameters on the thrust force. In addition, the interactions between the workpieces’ fibre orientation and the tool rake angle, and the depth of cut parameters, were also found to be significant. Figure 25a demonstrates the effect of tool rake angle on thrust force when machining samples with different workpiece fibre orientations. At CFRP at *θ* = 135°, a decrease in the thrust force as increasing the tool rake angle was observed. In particular, large tool rake angle led to increased thrust forces when machining workpieces with a fibre orientation of 45° at a cutting depth of 100 µm, as illustrated in Figure 25b. Figure 26 illustrates the impact of different combinations of the depth of cut and rake angle on the thrust force when cutting samples with fibre orientation of *θ* = 90°.

**Figure 27:**
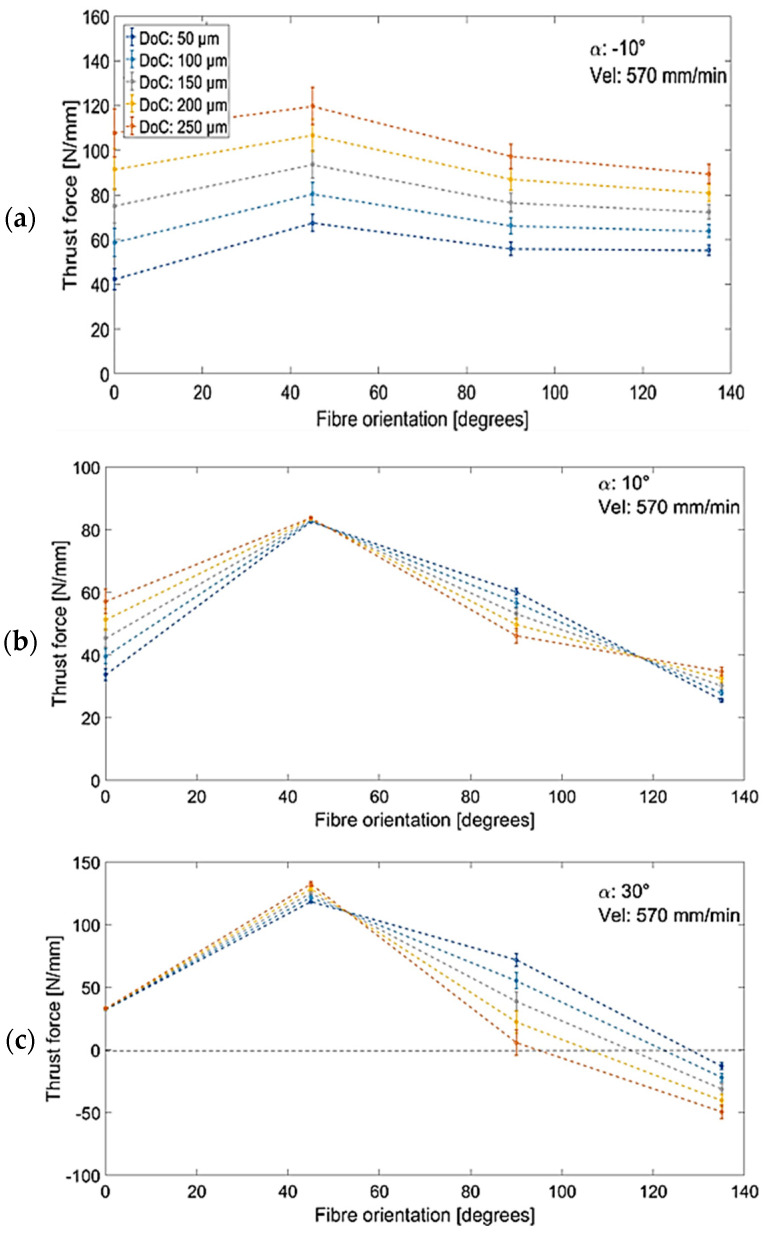
The trend of thrust force with respect to fibre orientation during cutting at a speed of 570 mm/min and rake angles of (**a**) −10°, (**b**) 10°, (**c**) 30°.

**Figure 28:**
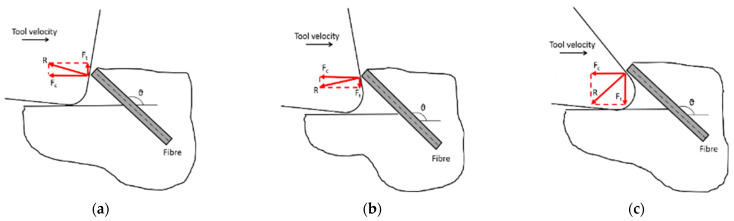
The thrust force component’s influence is schematically illustrated for (**a**) negative rake angle, (**b**) low positive rake angle, and (**c**) large positive rake angle.

Figure 27 illustrates the observed relation of the measured thrust force to the fibre orientation angle, tool rake angle, and cutting depth, including workpieces with a fibre orientation of *θ* = 0°. In the case of machining using tools with a negative rake angle (Figure 27a), a variation in thrust force of about 20 N/mm was noted. Furthermore, increasing the depth of the cut resulted in increasing the thrust forces, which is in agreement with Li et al. [23]. The relation for tools with positive rake angles (Figure 27b,c) is more complex. For the cutting tool with a rake angle of α = 30° (Figure 27c), a flip in thrust force direction was observed when machining workpieces with large fibre orientations. Similar characteristics were also reported in [6], which can be attributed by considering the schematic diagram in Figure 28. For tools with negative rake angles (Figure 28a), the thrust force component on the rake face is seen to push the tool upwards. As the tool rake angle varies from a negative to a high positive value (Figure 28c), the resultant force rotates counter clockwise, thereby producing a downwards thrust force. Therefore, tools featuring a negative rake angle tend to exert a downward pushing force on fibres, while tools with a positive rake angle pull the fibres upwards. However, the total thrust force must take into account other contributions, such as bounceback, which typically applies an upward force on the cutting tool.

### 3.8. Optimisation of Process Parameters

The optimal combination of machining parameters and output variables was determined using Design-Expert 7.0 software using a cutting depth of 100 µm. The targets are to minimise the cutting and thrust forces, reduce surface quality, and minimise the depth of workpiece damage. Different weightings were assigned to the outputs, with greater significance given to the workpiece surface roughness over machining forces [26,27]. The results of this analysis are outlined in Table 8. The findings indicate that, to achieve optimal results when working on workpieces with a fibre orientation angle of 45°, it is advisable to use a cutting tool featuring a negative rake angle at low cutting speed. On the other hand, when machining samples with fibre orientations of 90° and 135°, it is recommended to use tools with a significantly positive rake angle operating them at high cutting speeds to obtain the best results.

## 4. Conclusions

The study investigated the effects of workpiece fibre orientation and tool rake angle on chip morphology in orthogonal cutting of carbon fibre-reinforced polymer (CFRP). The results showed that increasing the rake angle of cutting tools favours continuous chip morphology, while fibre orientation strongly influences the chip formation mechanism. Negative rake angle tools can cause fibre buckling and bending, while low positive rake angles can result in fibre bending near the cutting edge and forming a continuous chip. Tools with high positive rake angle create a secondary cutting zone in front of the cutting tool, where chips are formed owing to the peeling action applied by the tool. The effect of rake angle is negligible at a fibre orientation of 45°. In contrast, for 90° orientation, high positive rake angle tools exhibit similar behaviour to 45°, and at 135°, mechanisms of chip formation are the same regardless of tool rake angle. The study also examined the effect of rounded cutting edges on chip formation mechanisms for workpieces with fibre angles of 0° and 90°. ANOVA results show that fibre orientation is the most significant factor affecting cutting and thrust forces, changing thrust force direction when machining at high fibre orientation and rake angles. Increasing fibre orientation angle and depth of cut leads to a higher damage depth, while larger tool rake angles generally reduce damage. Machining workpieces with *θ* = 0° results in the lowest subsurface damage, and surface roughness is independent of the tool rake angle for fibre orientation of 0° to 90° but deteriorates for fibre orientation larger than 90°. Combining tools with low positive or negative rake angles for samples with large fibre orientation of *θ* = 135° results in an elevated bounceback value due to material deformation below the cutter, which experiences elastic recovery after machining. Large rake angles and fibre orientation lead to a higher actual depth of cut with no bounce back observed.

## Figures and Tables

**Table 1:** Properties of fibre and matrix phases in UD-CFRP [19,20].

Material	Property
Carbon fibre—T800S	Fibre diameter: 5 μm
	Volume of fibre: 56.6%
	Elastic modulus: 294 GPa
	Tensile ultimate strength: 5.88 GPa
	Density of fibre: 1.80 g/cm^3^
HexPly^®^ M21	Elastic modulus: 172 GPa
	Tensile ultimate strength: 3 GPa
	Compression modulus: 136 GPa
	Compression ultimate strength: 1.67 GPa
	Shear modulus: 5 GPa
	Shear strength: 79 MPa

**Table 2:** Machining parameters applied to experiments.

Factor	Symbol		Levels	
Orientation of Fibre (degrees)	*θ*	0°	45° → 90°	135°
Cutting speed (mm/min)	$V_{c}$		12 → 570	1100
Rake angle (degrees)	*α*		−10° → 10°	30°
Depth of cut (µm)	DOC		50 → 100	150

**Table 3:** The orthogonal cutting experiments.

Orientation of Fibre	Rake Angle	Cutting Speed	Depth of Cut	Exp. No
(Degrees)	Degrees	(mm/min)	(µm)	
		12	50/100/150	1
	−10°	570	50/100/150	2
		1100	50/100/150	3
		12	50/100/150	4
*θ* = 0°	10°	570	50/100/150	5
		1100	50/100/150	6
		12	50/100/150	7
	30°	570	50/100/150	8
		1100	50/100/150	9
		12	**50**/100/**150**	10
	−10°	570	50/100/150	11
		1100	**50**/100/**150**	12
		12	50/100/150	13
*θ* = 45°	10°	570	50/**100**/150	14
		1100	50/100/150	15
		12	**50**/100/**150**	16
	30°	570	50/100/150	17
		1100	**50**/100/**150**	18
		12	50/100/150	19
	−10°	570	50/**100**/150	20
		1100	50/100/150	21
		12	50/**100**/150	22
*θ* = 90°	10°	570	**50/100/150**	23
		1100	50/**100**/150	24
		12	50/100/150	25
	30°	570	50/**100**/150	26
		1100	50/100/150	27
		12	**50**/100/**150**	28
	−10°	570	50/100/150	29
		1100	**50**/100/**150**	30
		12	50/100/150	31
*θ* = 135°	10°	570	50/**100**/150	32
		1100	50/100/150	33
		12	**50/**100/**150**	34
	30°	570	50/100/150	35
		1100	**50**/100/**150**	36

**Table 4:** Experimental results for RMS design.

Fibre Orientation (Degrees)	Rake Angle (Degrees)	Cutting Speed (mm/min)	Depth of Cut (mm)	Cutting Force (N/mm)	Thrust Force (N/mm)	Surface Roughness (Ra, µm)	Damage Depth (µm)	Bounce Back (µm)	N
		12	50	38.3	52.9	0.56	117.2	24	10
	−10	12	150	113.4	81.7	0.62	292	24	10
		1100	50	25.5	50.5	0.3	117	23.9	12
		1100	150	78.4	80.44	0.47	182.5	30	12
*θ* = 45°	10	570	100	60.2	82.9	1.02	328.5	16.1	14
		12	50	62.4	115	0.45	162.3	31.6	16
	30	12	150	102	122.7	1.37	328.5	36.4	16
		1100	50	61.63	117	0.54	122.55	20.7	18
		1100	150	90.14	124.7	1.11	255.47	23.59	18
	−10	570	100	93.64	66.27	1.06	55.235	34.7	20
		12	100	103.1	66.26	0.85	760.24	23.43	22
		570	50	78.5	60.2	0.52	245,098	8.4	23
		570	100	131.9	44.6	2.31	2043.8	19.54	23
*θ* = 90°	10	570	100	157.9	35.72	2.73	2549.02	28.18	37
		570	100	140.6	56.67	1.73	980,392	7.49	38
		570	150	202.7	53.17	2.81	2549.02	12.61	23
		1100	100	147.3	54.18	1.96	985.4	15.14	24
	30”	570	100	129.6	55.29	3.2	1240.88	29.25	26
		12	50	124	42.1	18.5	1372.55	194.159	28
	−10	12	150	211.7	72.46	29.16	4901.96	413.12	28
		1100	50	165	49.5	17.72	2745.1	159.98	30
		1100	150	200.5	72.87	43.29	4411.76	295.304	30
	10	570	100	214.2	27.88	19.02	2352.94	55.14	32
		12	50	189	-24	5.83	1960.78	0	34
	30	12	150	220.5	-37.2	14.7	3000	0	34
		1100	50	147	-20	6.11	1431.37	0	36
		1100	150	185.1	-29.9	14.74	2549.02	0	36

**Table 5:** Influence of process parameters and interactions on various response measures based on *p*-value. (bold numbers are those with less than 0.05).

Process Parameters	Cutting Force	Thrust Force	Damage Depth	Surface Roughness	Bounce Back
Fibre orientation ($x_{1}$)	**<0.0001**	**<0.0001**	**<0.0001**	**<0.0001**	**0.0004**
Rake angle ($x_{2}$)	0.1930	**0.0012**	0.1799	**0.0008**	**<0.0001**
Cutting velocity ($x_{3}$)	0.5353	0.8442	0.9714	0.3409	0.3438
Depth of cut ($x_{4}$)	**0.0001**	**0.0174**	**0.0013**	**0.0017**	0.0616
$x_{1}x_{2}$	0.8344	**<0.0001**	0.0783	**0.0003**	**<0.0001**
$x_{1}x_{3}$	0.8941	0.5964	0.9605	0.3111	0.4457
$x_{1}x_{4}$	0.9728	0.2285	**0.0141**	**0.0023**	0.0687
$x_{2}x_{2}$	0.4595	0.7523	0.4614	0.3628	0.4858
$x_{2}x_{3}$	0.2498	**0.0032**	0.2455	0.2198	0.0606
$x_{4}x_{4}$	0.4185	0.9422	0.4471	0.3058	0.6491
$x_{1}^{2}$				**0.0022**	0.3061
$x_{2}^{2}$				0.9083	0.3703
$x_{3}^{2}$				0.8241	0.6588
$x_{4}^{2}$				0.9205	0.9049

**Table 6:** Models’ summary statistics.

Output Variable	Model Fit	R-Squared
Cutting force	2FI	0.89
Thrust force	2FI	0.98
Damage depth	2FI	0.87
Surface roughness	Quadratic	0.95
Bounce back	Quadratic	0.91

**Table 7:** Model coefficients for each output variable.

Coeff.	Cutting Force	Thrust Force	Damage Depth	Surface Roughness	Bounce Back
a	−62.34078	71.16719	−1195.60251	20.61315	28.33589
b	1.27039	−0.34785	12.29852	0.63961	−1.43711
c	1.44578	3.82401	43.43755	0.39097	4.34509
d	0.012795	−3.61647 × 10^−3^	0.51158	−2.61787 × 10^−3^	−0.021271
e	0.75006	0.29579	−1.38042	-0.056215	−0.37485
f	−1.40208 × 10^−3^	−0.039019	−0.32295	−4.77887 × 10^−3^	−0.074506
g	3.28074 × 10^−5^	4.79473 × 10^−5^	3.17758 × 10^−4^	3.64457 × 10^−5^	−3.42804 × 10^−4^
h	−9.13889 × 10^−5^	−1.20889 × 10^−3^	0.18927	1.44424 × 10^−3^	9.45784 × 10^−3^
i	−4.13660 × 10^−4^	6.41085 × 10^−5^	−0.010718	−7.33613 × 10^−5^	7.03574 × 10^−4^
m	−7.09187 × 10^−3^	−7.51125 × 10^−3^	−0.18628	−1.09216 × 10^−3^	−0.022043
n	−1.81319 × 10^−4^	−5.88235 × 10^−6^	−4.42734 × 10^−3^	3.31766 × 10^−5^	−1.82604 × 10^−4^
p	0	0	0	4.01949 × 10^−3^	0.014014
q	0	0	0	6.19128 × 10^−4^	0.061777
r	0	0	0	−1.61521 × 10^−6^	4.06196 × 10^−5^
s	0	0	0	−8.57595 × 10^−5^	1.29633 × 10^−3^

**Table 8:** Optimal combinations of cutting velocity and rake angle for different fibre orientations at a constant depth of cut of 100 µm.

*θ*	*α*	Cutting Speed	$F_{c}$	$F_{t}$	Surface Roughness	Damage Depth
		(mm/min)	(N/mm)	(N/mm)	(µm)	(µm)
45°	−10°	366.82	62.78	65.49	0.3	38.53
90°	30°	1100	128.22	47.63	0.3	1102
135°	30°	1100	184.70	−23.76	12	2087

## Data Availability

The data presented in this study are available on request from the corresponding author. The data are not publicly available due to the extremely large size.
